# Increasing exercise frequency is associated with health and quality-of-life benefits for older adults

**DOI:** 10.1007/s11136-019-02264-z

**Published:** 2019-08-13

**Authors:** Kenneth P. Kell, Elizabeth Y. Rula

**Affiliations:** Tivity Health, 701 Cool Springs Blvd, Franklin, TN USA

**Keywords:** Seniors, Physical activity, Quality of life, SF-12, Healthy days, General health

## Abstract

**Purpose:**

To evaluate whether health-related quality-of-life measures can be improved in a senior population by increasing participation in an exercise program.

**Methods:**

The study involved a nationwide sample of adults aged 65 and older (mean age 73.2 in first study year) who participated in the SilverSneakers fitness program between 2010 and 2016. We analyzed data from 7 years of program participation records and annual participant surveys. Study members completed ≥ 2 annual surveys (*n* = 46,564). Participation frequency change was measured by average visits per week (AVPW) to a fitness center from the initial survey year to follow-up years. Quality-of-life measures included the 12-Item Short Form Health Survey (SF-12), Self-Rated Health, and BRFSS Healthy Days measures. Longitudinal analyses evaluated whether an increase in visit frequency among active members of SilverSneakers was associated with change in quality-of-life measures, controlling for age and gender.

**Results:**

Participants with more frequent visits (higher AVPW) had better SF-12 Physical and Mental Component Scores, Self-Rated Health Status, and fewer physically and mentally unhealthy days (*p* < 0.001 for all measures); furthermore, participants who increased AVPW longitudinally saw improvements in all outcome measures (*p* < 0.001).

**Conclusion:**

SilverSneakers participation frequency is associated with higher quality of life for seniors.

## Purpose

Americans 65 and older are expected to outnumber children by 2035 [1], a shift that has never occurred before in U.S. history, and by 2060, almost 1 in 4 Americans will be at least 65 years old [2]. These dramatic changes in demographics, driven in part by increases in life expectancy [3], underscore the important public health goal of maintaining and improving quality of life for aging populations [4, 5]. One pathway to improve quality of life may be via increasing one’s physical activity levels [6]. Physical activity has been shown to benefit various aspects of quality of life such as physical functioning, mental health, and social connection cross-sectionally, including in senior populations [7–13]. However, research that longitudinally evaluates whether increasing the frequency of older adults’ physical activity results in greater improvement in quality of life is sparse, particularly research that uses widely adopted metrics for health-related quality of life (HRQOL).

Health-related quality of life is an important metric to target as it is associated with mortality and hospitalizations in older adults [14–16]. Prominent efforts to identify and measure the factors that improve health-related quality of life in older adults in the US include the Healthy People 2020 goals [4, 5], the Medicare Health Outcomes Survey [17], and Medicare Part C (“Medicare Advantage”) Star ratings [18]. The purpose of this study is to longitudinally evaluate whether increasing participation frequency among members of a nationwide fitness program for older adults, SilverSneakers, is associated with improved HRQOL.

## Methods

SilverSneakers is a program that provides seniors with access to a nationwide network of over 15,000 fitness centers and a variety of specialized group exercise classes for older adults across the US.

The study population included seniors (65 years and older), who were members of SilverSneakers via their Medicare Advantage health plan, and who made at least 12 visits to a fitness center in the prior 12 months, with at least one visit in the prior 6 months. Participants had to complete at least two annual surveys from 2010–2016; however, to increase generalizability while maximizing evaluation of longitudinal outcomes, survey response years were not required to be consecutive. The total number of participants who met these criteria was 46,564.

We collected data via a recurring annual member survey (AMS) which is administered via a mailed paper survey in September every year. The average completion rate for this survey was 48% across the study timeframe. Included within the survey instrument were quality-of-life measures (SF-12, Self-Rated Health, and BRFSS Healthy Days measures), age, and sex. All measures were not available in every year of the annual survey: SF-12 items were assessed from 2010–2015, Self-Rated Health from 2010–2016, and Healthy Days from 2010–2011 and 2016.

The quality-of-life measures in this study are included in the surveys of major national initiatives. Healthy People 2020 quality-of-life measures include the BRFSS Healthy Days measure [19] and Self-Rated Health status [5] (SRH), a standalone item in the 12-Item Short Form Health Survey (SF-12) [20]. The SF-12, derived from the SF-36 used in the Medicare Health Outcomes Survey [17], is used to evaluate Medicare Part C (“Medicare Advantage”) Star ratings in the form of the Veterans RAND 12-Item Health Survey [18].

The scales for the quality-of-life dependent variables were SF-12 physical and mental component scores [20, 21], assessed on a scale from 0–100 with higher scores being better; Self-Rated Health [5] (“In general, would you say your health is:”), scored as 1–5 (“excellent” to “poor” with a higher number being worse); and Healthy Days which is the reported number of days in the past 30 days that mental and physical health were “not good,” [19], assessed as the number of unhealthy days, with a higher number being worse.

The primary independent variable was frequency of program participation, measured objectively by average visits per week (AVPW) to a SilverSneakers fitness location from recorded visits in internal accounting systems. AVPW was calculated on an annual basis for each calendar year that a participant completed a survey and evaluated as 4 tiers: < 1 visit per week, 1 up to < 2, 2 up to < 3, and 3 or more. “3 or more” was selected as the top tier of participation frequency as it echoes WHO exercise recommendations [22], and exercising at least 3 times per week has shown to be a threshold for positive impacts on mental health [23].

Statistical tests of the cross-sectional association between AVPW and quality-of-life measures used the Friedman test as there are more than two tiers and they are not necessarily independent (the same participant can be in different tiers of AVPW over time).

Longitudinal analyses were performed using generalized linear modeling utilizing generalized estimating equations, controlling for age and sex. Data were first transformed into long form by year, and the participant ID was identified as the class statement and the repeated subject. Since most of the participants did not have more than three time points in which they had survey responses, the model was run with an unstructured correlation matrix.

All analyses were conducted using SAS Enterprise Guide 7.1

## Results

**Fig. 1:**
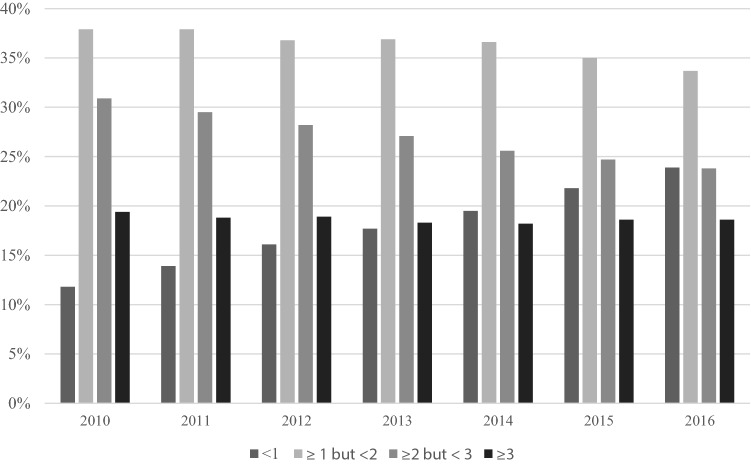
Percent of population by tier of average visits per week, by year

Descriptive statistics by year are presented in Table 1 and percentages by AVPW by year are shown in Fig. 1. Evaluation of the relationship between AVPW and each outcome variable found that more frequent visits to a fitness center were associated with significantly better outcomes across all quality-of-life measures (Table 2; *p*-values < 0.001).

**Table 1:** Descriptive statistics for control and dependent variables by year

Variable	Year	N	Mean	Std. Dev.
Age	2010	15,895	73.22	6.06
	2011	21,347	73.93	6.23
	2012	21,730	73.73	6.38
	2013	20,350	73.66	6.58
	2014	18,146	73.45	6.57
	2015	14,217	74.13	6.49
	2016	3697	75.01	6.64
Percent female	2010		62%	
	2011		62%	
	2012		61%	
	2013		60%	
	2014		59%	
	2015		58%	
	2016		57%	
SF-12: physical component score	2010	14,437	48.55	8.90
	2011	18,282	47.99	9.35
	2012	19,602	48.22	9.34
	2013	18,269	48.43	9.24
	2014	18,126	48.45	9.20
	2015	12,774	48.39	9.14
SF-12: mental component score	2010	14,437	55.70	6.88
	2011	18,282	55.50	7.04
	2012	19,602	55.52	7.09
	2013	18,269	55.55	7.08
	2014	18,126	55.58	7.06
	2015	12,774	55.65	6.99
Self-rated health	2010	15,831	2.29	0.78
	2011	21,133	2.32	0.79
	2012	21,509	2.30	0.79
	2013	20,161	2.29	0.79
	2014	19,767	2.27	0.80
	2015	14,086	2.27	0.79
	2016	3660	2.31	0.81
Physically unhealthy days	2010	14,155	2.52	6.15
	2011	19,966	2.32	6.05
	2016	3173	2.79	6.96
Mentally unhealthy days	2010	14,248	1.76	4.92
	2011	20,175	1.57	4.55
	2016	3302	1.47	5.01

**Table 2:** Descriptive statistics per AVPW (average visits per week) for all dependent variables across all available data

	AVPW	N	Mean	Std Dev
SF-12: physical component score*	< 1	13,659	47.05	9.79
	≥ 1 but < 2	38,104	47.75	9.42
	≥ 2 but < 3	29,412	48.72	8.94
	≥ 3	20,133	49.72	8.57
SF-12: mental component score*	< 1	13,659	54.84	7.65
	≥ 1 but < 2	38,104	55.52	7.10
	≥ 2 but < 3	29,412	55.75	6.79
	≥ 3	20,133	55.94	6.74
Self-rated Health^a^*	< 1	15,898	2.39	0.81
	≥ 1 but < 2	43,551	2.32	0.79
	≥ 2 but < 3	33,272	2.26	0.77
	≥ 3	22,829	2.20	0.79
Physically unhealthy days^a^*	< 1	4640	3.33	7.19
	≥ 1 but < 2	14,071	2.64	6.39
	≥ 2 but < 3	11,134	2.13	5.70
	≥ 3	7116	1.87	5.47
Mentally unhealthy days^a^*	< 1	4720	2.01	5.32
	≥ 1 but < 2	14,198	1.67	4.72
	≥ 2 but < 3	11,269	1.54	4.53
	≥ 3	7186	1.41	4.52

The sample size for each tier of AVPW can exceed the sample size for any given year because the analysis aggregates data from all years and participants are represented at least twice in the dataset

^a^For these measures, a smaller number signifies better health

*Results from the Friedman test indicate that mean values differ by tier of AVPW (*P* value < 0.001)

In longitudinal analyses, increasing frequency of visits from baseline to follow-up was associated with significant improvements in all quality-of-life-dependent variables analyzed, with all *p*-values falling below 0.001 (Table 3). For each incremental increase in AVPW, on average, SF-12 physical component score increased by 0.75 and mental component score increased by 0.33, Self-Rated Health decreased by 0.05, and physically and mentally unhealthy days decreased by 0.47 and 0.15, respectively.

**Table 3:** Longitudinal analyses using generalized linear modeling with generalized estimating equations

Measure	*P*-value	Effect size estimate	Average annual value
sf-12: physical component score	< .0001	0.75	48.33
SF-12: mental component score	< .0001	0.33	55.57
Self-rated health^a^	< .0001	− 0.05	2.29
Physically unhealthy days^a^	< .0001	− 0.47	2.44
Mentally unhealthy days^a^	< .0001	− 0.15	1.63

^a^For these measures, a negative estimate signifies better health

Therefore, for each incremental tier increase in AVPW, on average, SF-12 physical and mental component scores increased by 1.5% and 0.6% respectively, Self-Rated Health improved by 2.4%, and physically and mentally unhealthy days decreased by 19.4% and 9.5%, respectively.

## Discussion

Decreasing quality of life has typically been associated with the aging process, but these declines are in large part attributable to the consequences of decreasing physical activity [24, 25]. Therefore, increasing one’s physical activity should result in increased quality of life. This study indicates that even in older adults who are already physically active, quality of life may be further enhanced by increasing participation in SilverSneakers. For example, on average, an increase from less than 1 fitness center visit per week to at least 3 visits per week was associated with a reduction in physically unhealthy days of more than 1.4 days per month, and a reduction in mentally unhealthy days of almost 0.5 days per month. Not only do reductions in unhealthy days positively impact quality of life for the individual, but research by a major Medicare Advantage health plan provider has estimated a healthcare cost of $15.64 for each additional unhealthy day per person per month [26], further demonstrating the value of reducing unhealthy days.

The significant longitudinal effect observed for each of the study measures may also have clinical significance. In particular, the SF-12, Self-Rated Health, and unhealthy days measures predict hospitalizations and mortality, suggesting that increasing participation in SilverSneakers may influence these outcomes. The potential for this clinical impact is supported by prior research that demonstrated the relationship between SilverSneakers participation and hospitalizations [27, 28].

This study used a large sample, followed across multiple years, but there were some limitations. Survey responses were not available for every year and were not always contiguous. Also, due to a change in survey sampling in 2016, later years had a smaller useable sample size. Since the study was retrospective in nature and not originally structured to include a control group, there was not one available for inclusion in analyses. Finally, as the participants were not observed by the researchers during fitness center visits, it is not possible to attribute results directly to physical activity (e.g., intensity or duration), and other aspects of the program, such as socialization with peers, may have contributed to the observed relationship between visits and HRQOL. We posit that the primary driver of this relationship is higher physical activity levels, supplemented by the program’s social benefits, as was observed in a recent cross-sectional study of SilverSneakers members [29].

The survey sample for this study was limited to older adults with at least some physical activity, whereas 28% of adults 50 years and older are physically inactive [30]. Future research should focus on a cohort that increases physical activity from a baseline of no regular exercise. An inactive cohort would likely demonstrate a much larger effect size of increasing activity since the benefits of exercise on health and parameters of quality of life are most pronounced when someone moves from being physically inactive to engaging in some level of activity [31, 32], congruent with a study demonstrating SF-12 physical and mental differences between older adult exercisers and non-exercisers [33].

Overall, this study supports that increasing participation in the SilverSneakers fitness program is an effective strategy to improve health-related quality of life among older adults. These results show that it is never too late to improve one’s quality of life, nor are the benefits of increasing exercise frequency reserved for those of any particular age.
